# Profiles of Health-Related Patient Activation and Their Determinants: The Results of a Cluster Analysis of Older Adults—Conclusions for Patient Counselling

**DOI:** 10.3390/ijerph19042487

**Published:** 2022-02-21

**Authors:** Dorota Włodarczyk, Joanna Chylińska

**Affiliations:** Department of Health Psychology, Medical University of Warsaw, 00-575 Warsaw, Poland; dorota.wlodarczyk@wum.edu.pl

**Keywords:** patient activation, older adults, patients expectations, proactivity profile

## Abstract

Health-related proactivity in older adults may significantly increase medication handling, adherence and patient safety. Deficiencies in training in critical characteristics and diversity of older patients may lead to medical errors in diagnosis and drug administration. This study investigated the profiles of health proactivity in older adults and the factors differentiating them, like sociodemographic factors, health status, visit characteristics, and patients’ visit-related expectations, actual experiences, and satisfaction with the visit. Before and after visits, 3391 patients aged 65–95 filled in two sets of questionnaires, that allowed to measure aforementioned factors. Three distinct proactivity profiles emerged from a cluster analysis: high (43%), medium (25%), and low proactivity (32%). Highly proactive patients had the highest expectations, but their visits provided better opportunities to meet them than in other groups. Higher proactivity was related to a longer attendance time, frequent contact with and easier access to the doctor, or a longer time spent with a patient. The findings highlight the need to detect and respond to patients’ expectations regarding psychosocial aspects of care, as well as to improve organizational aspects of care, in order to enhance health proactivity in older adults. The resulting good practice recommendations may significantly improve healthcare workers’ effectiveness in both primary and secondary care.

## 1. Introduction

Demographic changes, the prevalence of chronic health conditions, and older adults’ increasing use of health services [1] generate considerable challenges for healthcare systems. Research has recognized patient activation (PA), encouraging patients to participate in their own care, as a way to improve individual and population health and reduce costs [2]. Higher PA produces self-management [3,4], health-related behaviours [5,6], the use of screening services and preventive behaviours [5,7,8,9,10], and a reduction of healthcare-related costs [5,11]. Health-related proactivity in older adults may significantly increase medication handling, adherence, and patient safety in everyday clinical practice [12]. Low PA has also been related to increased risk of hospitalization and Emergency Room utilization [13].

PA refers to patients’ knowledge, skills, and confidence in managing their own health and care [12,14]. Usually, studies measure it unidimensionally and categorize it on four levels from passive to proactive [15]. Following a clinical approach, we used the multicomponent concept of attitudes towards treatment and health (ATH). It encompasses cognitive, emotional, and motivational–behavioural dimensions. The cognitive aspect refers to patients’ health knowledge and expectations regarding one’s health and healthcare; the emotional component, to emotions experienced by patients in regard to their health situation (we analysed the level of positive and negative emotions separately); and the motivational-behavioural component, to plans and actions undertaken to manage one’s health [16,17]. As the PA concept includes patients’ confidence, the dimension of self-efficacy has also been included [18,19,20,21]. This approach involves presenting the level of PA as a profile of results in the five aforementioned dimensions. Based on the ATH, health-proactive patients understand their health situation, respond to health challenges with adequate emotions, have the motivation to plan and maintain health activities, and feel confident about their successful self-management of health goals. This more complex description of PA can enhance the quality of clinical interventions by indicating which aspects of the ATH require modification to increase PA. Deficiencies in training regarding critical characteristics and diversity of older patients may lead to medical errors in the diagnosis and drug administration [22,23]. Poor adherence is one of the significant issues in the group of older adults, as they experience the highest burden of chronic diseases, and subsequently, polypharmacy and regimen complexity [24,25].

Research has indicated that PA is especially low among patients who are chronically ill or suffering from multi-comorbidity [8,26,27,28], and interventions to enhance it are not particularly effective [26]. Although PA appears to be modifiable [29], the predictors of positive change [30] and the determinants of activation in older adults remain unclear.

Studies of such predictors have focused mainly on sociodemographic and clinical factors [10,31]. However, some research has indicated that patient-centered care may also play a role [32]. Studies have found that meeting older patients’ expectations, empowering them in the process of decision-making, effective communication, and satisfaction with a visit are directly or indirectly related to more favourable outcomes [33,34,35]. It is not clear, however, how the level of proactivity relates to patients’ visit-related expectations and actual experience.

Our first aim was to study health proactivity profiles among older adults. Our second aim was to compare these profiles in terms of sociodemographic factors, the health status and visit characteristics, as well as patients’ visit-related expectations and patients’ experiences during the visit and overall satisfaction with it. Among patients’ visit-related expectations, we distinguished the needs to know and to feel understood and acknowledged [36]—in short, the knowledge, support, and rapport triad (KSR triad)—and those related to doctors’ communication skills in the form of specific behaviours and techniques.

## 2. Materials and Methods

### 2.1. Participants and Procedure

The participants of the multi-site study were 3391 patients aged 65–95 (M = 73.58; SD = 6.27) attending 151 primary care facilities (PCFs) in Central Poland, with funding from the National Health Fund, within the PRACTA Promoting Active Ageing project (www.practa.wum.edu.pl (accessed on 18 December 2021). Data were collected between October 2013 and March 2014. The sample size we investigated was derived from the number of doctors participating in the project (approx. 10 patients per doctor) [19]. We performed simple randomization in selecting PCFs, obtaining a 20% response rate (151 out of 767 invited facilities). Trained interviewers interviewed the patients in the facility waiting room before and after the visit (when checking for missing data as a part of the procedure). The inclusion criteria were age 65+, having an appointment on the given day with a GP recruited for the project (50% of invited GPs agreed to participate), the ability to fill in questionnaires independently, and consent to participate. The patient response rate was 76% (we did not collect data on reasons for refusal). The institutional bioethics committee approved this study (ref. no KB/10/2014; 14 January 2014).

### 2.2. Measures

Before visits, patients responded to the PRACTA Patient Expectations Scale–Pre (PES-Pre), the PRACTA Communication Scale–Pre (CS-Pre), and a survey measuring socio-medical factors. After visits, the assessment included the PRACTA Satisfaction with Visit Scale (SVS), the PRACTA Patient Experiences Scale–Post (PES-Post), the PRACTA Communication Scale–Post (CS-Post), the PRACTA Attitude towards Treatment and Health Scale (ATH), and the PRACTA Self-Efficacy Scale (S-ES). All measures were developed and adapted within the PRACTA project, and their full versions are available at www.practa.wum.edu.pl (accessed on 18 December 2021), as well as in the Supplementary Materials. Psychometric properties of all tools applied in the study were investigated and found to be satisfactory [20,37,38]).

We used the PES-Pre to measure patients’ pre-visit expectations, whereas the PES-Post assessed their post-visit experiences. The scales contained 18 identical items but differed in their instructions: ‘During this visit it’s important to me, that the doctor …’ for the PES-Pre, and ‘During this visit the doctor …’ for the PES-Post. Patients responded using a seven-point Likert scale. Both scales consist of six three-item subscales concerning expectations of disease explanation, treatment explanation, emotional support, health promotion, quality of life improvement, and rapport. The Cronbach’s alphas for the subscales vary between 0.83 and 0.95 [37].

CS-Pre and the CS-Post respectively assessed patients’ expectations regarding doctors’ communication skills and evaluation of doctors’ actual communication. The scales consist of 26 identical items specifying GPs’ communication behaviours. They differed in instructions: ‘It’s important for me that the doctor …’ (e.g., greets me in a kind manner) for the CS-Pre and ‘During this visit the doctor …’ (e.g., greeted me in a kind manner) for the CS-Post. The patients responded using a seven-point Likert scale. Both reliability coefficients were α = 0.96 [37].

The collected medical data included the following indices of health status: number of diseases treated, self-rated health (SRH; reverse scale), use of healthcare within the last 6 months, reasons for the current visit, and health impact on activities of daily living (HIA), comprising 10 everyday activities evaluated on a four-point scale, from one (doesn’t limit at all) to four (limits very much) (reliability coefficient α = 0.95) [37,38].

The Satisfaction with Visit Scale (SVS) consists of seven items (e.g., ‘Would you recommend this doctor to your family/friends?’) scored on a seven-point scale (reliability coefficient α = 0.93 [37,38]). The interviewers measured the length of the visit. Organizational aspects of care, such as length of attendance with the same practitioner, seeing the same practitioner within the previous year, waiting time from registration and difficulty of registrations were also asked for.

The ATH and the S-ES measure health proactivity and have the same instruction and format. The ATH contains 16 items and has a four-factor structure that confirmatory factor analyses have confirmed [20], representing the following components: cognitive, emotional–positive, emotional–negative, and motivational. S-ES has three items, creating one scale. The scales begin with the same statement: ‘Due to this visit at the doctor …’–followed by individual items, for example, ‘I feel calmer’ (ATH emotional–positive scale) or ‘I think I can influence how I’ll feel in the future’ (S-ES). The patients responded using a seven-point Likert scale. A higher score reflects the greater intensity of an ATH dimension (including negative emotions). The reliability coefficients are 0.89 for the S-ES and between 0.89 and 0.92 for the ATH subscales [38].

### 2.3. Statistical Analyses

We verified the distribution of the variables with the Kolmogorov–Smirnov test. To establish proactivity profiles, we performed *k*-means clustering, a non-hierarchical cluster analysis, which allows to partition n observations into k-clusters, based on their similarity. That means that no predefined criteria of observations (results) are given. Patients were grouped into clusters depending on the similarity of the analyzed variables: four ATH scales and S-ES. The K-means algorithm assigns each point to the cluster whose center is nearest (this is classification criteria). The result of cluster analysis is the classification of cases into groups that are relatively homogeneous within themselves and relatively heterogeneous between each other [39]. We run one-way ANOVA to confirm that clusters differ significantly on all variables constituting them (all *p*-values < 0.001). The results we obtained show that H-pro scored above 6 on ATH-cognitive, ATH_motivation, ATH_Positive emotion and Efficacy, and around 2 on ATH-negative emotions. We observed that the L-pro group obtained scores of 4 or lower on ATH-cognitive, motivation, positive emotions, and S-ES, and above 5 on ATH-negative emotions We tested two-, three-, and four-cluster solutions and finally chose the three-cluster solution, as it allowed us to keep the sizes of clusters meaningful; we lost less information than in the two-cluster solution and achieved less complexity and greater applicability than in the four-cluster solution. To compare groups, we used one-way analysis of variance (ANOVA) for continuous, normally distributed variables (with Tamhane’s post hoc test), the Kruskal–Wallis test for ordinal or not normally distributed variables, and the chi-square test for categorical variables. We conducted the statistical analyses with SPSS version 20.0.

## 3. Results

### 3.1. Characteristics of Health Proactivity Profiles

**Figure 1:**
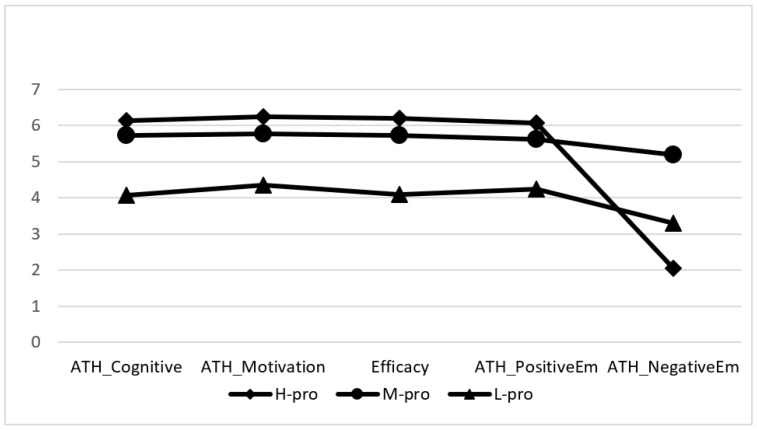
Profiles of health proactivity (*n* = 3391).

Based on the results of the four ATH subscales and S-ES scale, we distinguished three patient profiles: high (*n* = 1463, 43%), medium (*n* = 832, 25%), and low (*n* = 1096, 32%) proactivity (H-pro, M-pro, and L-pro, respectively) (Figure 1). The groups differed significantly in all aspects of proactivity. The H-pro reported the highest levels of health knowledge about their health, positive emotions, motivation, and self-efficacy and the lowest levels of negative emotions. The M-pro demonstrated the highest level of negative emotions and medium levels of other aspects of proactivity. The L-pro demonstrated a medium level of negative emotions and the lowest levels of other elements of proactivity.

### 3.2. Differences between Health Proactivity Profiles according to Sociodemographic Factors

As Table 1 shows, the profiles did not differ according to gender. The H-pro and L-pro groups did not differ in age, but both were significantly older than the M-pro group. The H-pro group had the largest percentages of single, divorced, and widowed people and people who lived alone. The H-pro group more frequently lived in large towns (above 500,000) or in the capital, whereas the L-pro group lived in rural areas and medium towns (101–500,000). The L-pro group also included the highest number of less educated patients. Most retired people belonged to the H-pro group, whereas slightly more still-working patients were in the L-pro group. Most of the unemployed were in the M-pro group. The H-pro group declared the best economic status, followed by the M-pro and L-pro groups.

### 3.3. Differences between Health Proactivity Profiles according to Health Status, Reasons for the Current Visit, and Satisfaction with the Visit

As Table 2 shows, the H-pro and L-pro patients did not differ in the number of reported diseases; however, the M-pro group had a significantly higher number. The H-pro group contained slightly more patients with two to four diseases, whereas the L-pro group had more patients without any chronic disease undergoing treatment. However, in the last 6 months, the L-pro group had used healthcare services more frequently than the other groups. The H-pro patients declared the best SRH, and the L-pro group the worst. Regarding the HIA evaluation, the M-pro group reported the least severe impairment than the H-pro group, and the L-pro group had the most severe impairment.

The H-pro group contained the highest percentage of patients whose aim of the visit was related to current treatment (medical advice or prescription of medications), whereas in the L-pro group, slightly more reported some formal aspects of treatment (referrals or formalities). More patients for whom this was the first visit to this GP were in the L-pro group. The H-pro group had a longer history of attending their GP than the other groups. Members of the H-pro group had visited their GPs most frequently in the past year, followed by the M-pro and L-pro groups. The waiting time for the visit after registration was the longest in the M-pro group; the other groups did not differ. The L-pro group reported the most difficulty getting to the GP, then the M-pro and H-pro groups. The H-pro group had the longest visits, with no differences between the M-pro and the L-pro group.

The global satisfaction with the visit was the highest for the H-pro patients, followed by the M-pro and L-pro patients (Table 2). The M-pro group was the least willing to recommend the GP (F = 3.06; *p* = 0.047); the H-pro and L-pro groups did not differ. Visiting the evaluated GP again and satisfaction with the visit length produced similar results (F = 5.02; *p* = 0.007 and F = 3.36; *p* = 0.035, respectively).

### 3.4. Differences between Health Proactivity Profiles according to Pre-Visit Expectations and Visit Experiences

Table 3 shows that the H-pro group had the highest pre-visit expectations regarding the KSR triad and doctors’ communication skills, with the exception of emotional support. They were also higher in the L-pro group than in the M-pro group, with the exception of disease explanation.

After the visit (Table 3), the L-pro group reported the lowest fulfilment of these expectations, with the exception of disease explanation. Regarding disease and treatment explanation, quality of rapport, and communication skills, the H-pro group reported greater fulfilment than the M-pro group; in cases of emotional support and health promotion, there were no differences; and the M-pro group declared the greatest fulfilment for quality of life.

The last part of Table 3 presents the discrepancy between expectations and experiences during the visit, understood as the difference between post- and pre-measurements. A bigger absolute value indicates a bigger discrepancy. The H-pro group showed the smallest discrepancy, and the L-pro group the biggest.

## 4. Discussion

We distinguished three health proactivity profiles among older adults. Through cluster analysis, we obtained profiles based on configurations of five ATH components. Detailed and empirically based characteristics of proactivity profiles are an alternative to unidimensional approaches to PA [13,28], and an added value of this research. H-pro patients constituted 43% of the sample, which is similar to the adult population [10] or comorbid diabetes and chronic kidney diseases [31] and lower than in hypertensive older patients [33] or frequent healthcare users [40].

The proactivity groups were similar between genders, replicating the existing evidence [29,30]. The H-pro patients were usually unmarried, living alone in big towns, retired, with higher education, and with a better economic status, in line with research on the adult population [10,31]. Therefore, it is worth highlighting that patients with a lower education level, socioeconomical status and living in smaller towns/rural areas would require more attention in terms of supporting their proactivity by doctors. The H-pro and L-pro groups were equally old, which was surprising and rather at odds with the other results [10,30,31].

Despite more H-pro patients reporting multiple morbidities and noticeable impairment of functioning, they had the best SRH. Although these results only partially align with other studies [30], they confirm that SRH is a meaningful correlate of proactivity. The L-pro group, having similar numbers of diseases to the H-pro group, used health services more frequently. This suggests that it is not the numbers of diseases, but their course and treatment that are crucial for proactivity. H-pro patients could also be more efficient in disease management [41], in turn leading to better SRH. Further research is necessary to determine whether the burden of disease contributes to low proactivity or vice versa.

The study showed that some formal and organizational aspects of care, like longer time of attendance, frequent contact with and easier access to the doctor, or longer time spent with a patient, accompany higher proactivity, suggesting the promotion of PA from the facility organizational level. These results are in line with prior evidence [42]. Explaining the mechanism of this relationship requires further research, as there are various possible explanations. One of them is that longer time of attendance is likely to relate to higher trust and acceptance of the doctor’s approach. This may favor compliance/adherence/concordance behaviours [43]. On the other hand, accessibility to healthcare (the patient is able to make an appointment at the time needed and at the preferred doctor, and therefore is able to maintain a longer time of attendance with the same doctor) would also be of importance. Additionally, further research should verify the visit length’s role, as it could relate to the visit’s aim; in the H-pro group, it was more frequently for treatment than formalities.

The H-pro group generally had the highest pre-visit expectations regarding the KSR triad and doctors’ communication skills, which suggests that the most demanding patients are also the most promising in health proactivity. This may sound controversial in the context of healthcare providers’ work overload when satisfying patients’ expectations may seem too time-consuming. Changing this misconception may help doctors to use older adults’ potential for proactivity better.

It seems that satisfying patients’ expectations is a precondition for activation. This study’s strength relied on capturing not only the subjective evaluation of the visit but also the discrepancy between pre- and post-visit evaluations. It confirmed that greater satisfaction with expectations is positively associated with the activation level [44]. This means that doctors spent the most time with and responded the most adequately to the needs of patients with the highest expectations. This could be the consequence of these patients’ more demanding and self-directing behaviour [32], but also of the fact that the most proactive group was better educated and better off, with good SRH despite the number of diseases. The latter might motivate doctors to engage more in the hope that the invested effort will benefit the patients and generate work satisfaction.

According to the transactional hypothesis, being an active patient affects interactions with healthcare providers, enhancing the care received [45]. The selection hypothesis states that more proactive patients choose providers who are able to give more patient-centred care. So far, more evidence supports the first hypothesis [44], showing that more active patients had more positive experiences than less active ones with the same clinician, even when controlling for demographic characteristics and health status [46]. Our results also support the notion that proactive patients have more resources to shape doctor–patient interaction in the desired direction and elicit what they need from their providers.

In clinical practice, a better understanding of the factors differentiating the health proactivity profiles may support physicians in identifying potential for improvements and prioritizing resource allocation to increase the quality of healthcare. For this purpose, other researchers proposed measuring activation as an intermediate outcome of care [46].

Our results show that older adults’ proactivity can be enhanced both by doctors providing patients with individualized assistance to self-manage their health and by improving organizational aspects of care and equity [47]. Further research is necessary on better integration of these two areas of the health system.

Promoting proactivity requires specific counselling skills, the lack of which is a serious barrier named by the doctors themselves [25]. From the clinical perspective, it is important to know, which information provided by patients during medical consultations are likely to predict levels of proactivity, and therefore, should catch the doctor’s attention. Subsequently, on the base of our results, we propose a three-step protocol, which summarizes key areas for investigation and interventions.

The first step carried out at the beginning of consultations (during the recognition of patient’s perspective stage) is to recognize the patients’ approach in two critical areas: KSR triad and SRH. The KSR triad includes information about the importance of the following aspects for the patient, such as the following. (1) Knowledge: to get necessary information (on disease/results of tests, treatment/medication, health promotion); (2) support: to talk about patients coping with the disease and quality of life improvement; (3) rapport: assuming that all patients expect to be treated seriously and with respect, it is important to evaluate the level of need for closeness and intimacy (rather on the base of non-verbal communication, as direct question can to too embarrassing). The second area regards SRH, that is, how, in comparison with people of the same age, a patient evaluates their health.

The second step includes responses by the doctor to detected needs, and it takes place throughout the visit. It encompasses providing expected information, building rapport and giving support as needed.

The final, third step, carried out before the stage of closing the consultation, refers directly to the diagnosis of the achieved level of proactivity. It includes checking for information on: how accurately does the patient understand their disease and treatment? Does the patient feel anxious or depressed? Does the patient believe that the treatment will be effective? Does the patient feel he/she will be able to implement given recommendations? Is the patient ready to comply with them?

Application of the aforementioned protocol, except understanding its content, also requires proficiency in communication. Describing appropriate communication methods extends the scope of the presented manuscript; however, we would like to point to scaling as a tool, which is simple and useful in practice. Allowing patients to choose their answer to the questions on the given scale (i.e., from 1—very poor, to 5—very good) helps to establish the level of proactivity—the higher the score, the more proactive the patient is.

Additionally, it is recommended to monitor the level of the visit satisfaction carried out outside of the doctor’s office, with the question regarding the degree of meeting expectations regarding this specific visit.

The study has some limitations. It only collected self-descriptive data from patients. Other health caregivers’ perspectives would be valuable to enhance the reliability of PA evaluation. Selection bias might have also affected patient recruitment, as only half of the doctors agreed to participate in the project. They could have been the doctors who had a more positive approach or were better trained in older adults’ care. Consequently, these could have been the patients who received especially good-quality care, perhaps better than average. The group consisted of older adults with a relatively high level of independence. Further research should include patients requiring more advanced or institutional care. This is especially important in the context of the risk of cognitive impairment in older adults, which this study did not assess directly. The only indicator of cognitive ability was the ability to complete questionnaires and use healthcare independently. Patients with reduced self-criticism accompanying dementia may overestimate their proactivity and quality of care [48]. Future research should include the diagnosis of cognitive functions and patients’ ability to express their needs [49]. The expectations of institutionalized older patients remain underdiagnosed and untreated [50]. Future studies should also verify the relationship between global and disease-specific PA and determine which of them to use in successful interventions and how. Finally, it should be mentioned that the study was conducted in the pre-pandemic period, and new demands like lockdown or e-consultation might influence older patients’ activation levels. Considering aforementioned factors, generalizability of our results and conclusions is limited to community-dwelling, older adults in the context of traditional (pre-pandemic) healthcare.

## 5. Conclusions

The results obtained in the study allow to conclude that older patients, aged 65+, whose visit-related expectations have been detected and responded to most accurately by physicians, report the highest levels of health proactivity. Additionally, patients who report highest health proactivity were simultaneously the most demanding—they declared the highest visit-related expectations. Improving organizational aspects of care, such as accessibility and continuity of medical care, may lead to enhanced health proactivity in older adults.

## Figures and Tables

**Table 1:** The differences between health proactivity profiles according to sociodemographic factors.

Factor	Level	H-pro	M-pro	L-pro	Test (*p*)
Age (M; SD)		73.65 (5.88)	72.92 (6.41)	74.16 (6.51)	F = 9.44 (<0.001) H > M; H = L; M < L *
Gender, *n* (%)	women	860 (59)	495(60)	656 (60)	chi^2^ = 0.31 (0.85)
	man	603 (41)	337 (40)	440 (40)	
Marital status	single	86 (6)	50 (6)	31 (3)	chi^2^ = 91.16 (<0.001)
	married	664 (45)	459 (2755)	613 (56)	
	divorced	136 (9)	42 (5)	24 (2)	
	widowed	577 (40)	281 (34)	428 (39)	
Cohabitation	alone	597 (41)	211 (25.8)	222 (20.6)	chi^2^ = 140 (<0.001)
	family	857 (58.8)	613 (74)	866 (79)	
	institution	3 (0.2)	2 (0.2)	4 (440.4)	
Place of living	rural area	90 (6)	54 (6)	180 (16)	chi^2^ = 283.63 (<0.001)
	below 20,000	53 (4)	67 (8)	61 (6)	
	between 21–100,000	170 (12)	131 (16)	102 (9)	
	between 101–500,000	14 (1)	9 (1)	51 (5)	
	above 500,000	457 (31)	182 (22)	120 (11)	
	capital	679 (46)	389 (47)	582 (53)	
Education	primary	139 (10)	90 (11)	199 (18)	chi^2^ = 128.31 (<0.001)
	vocational	577 (39)	252 (30)	366 331)	
	Secondary (no diploma)	219 (15)	102 (12)	215 (20)	
	Secondary (diploma)	329 (22)	235 (28)	235 (22)	
	higher	199 (14)	153 (19)	81 (7)	
Professional status	retired	1342 (92)	703 (85)	947 (86.8)	chi^2^ = 44.10 (<0.001)
	working	104 (7.6)	106 (13)	128 (12)	
	unemployed	6 (0.4)	18 (2)	9 (0.2)	
Economic status (M;SD)		3.11 (0.79)	3.00 (0.86)	2.78 (0.70)	BF = 57.05 (<0.001) H > M; H > L; M > L *

ATH_Cognitive—Cognitive Scale of ATH, ATH_Motivation—Motivational Scale of ATH, ATH_PositiveEm—Emotional-Positive Scale of ATH, ATH_NegativeEm—Emotional-Negative Scale of ATH. * Between-group comparisons with T2 Tamhane test.

**Table 2:** Differences between health proactivity profiles according to health status, reasons for the current visit, and satisfaction with the visit.

Factor	Level	H-pro	M-pro	L-pro	Test (*p*)
Number of diseases (M; SD)	-	1.88 (0.88)	1.62 (0.86)	1.97 (1.87)	F = 23.58 (<0.001) H > M; H = L; M < L *
Number of diseases, *n* (%)	none	75 (5)	62 (7)	93 (8)	chi^2^ = 89.09 (<0.001)
	1 disease	406 (28)	313 (38)	337 (31)	
	2–3 diseases	634 (43)	362 (44)	392 (36)	
	4 or more	358 (24)	95 (11)	274 (25)	
Health service within last 6 months, *n* (%)	no	1322 (90)	743 (89)	825 (75)	chi^2^ = 127.85 (<0.001)
	yes	141 (10)	89 (11)	271 (25)	
SRH (M; SD)	-	2.89 (0.66)	3.02 (0.82)	3.16 (0.68)	43.38 (<0.001) H < M;H < L; M < L *
HIA global	-	1.74 (0.74)	1.57 (0.69)	1.96 (0.81)	66.17 (<0.001) H > M; H < L; M < L *
Aim of the visit, *n* (%)	treatment	1389 (95)	752 (90)	999 (92)	chi^2^ = 24.46 (<0.001)
	formal	73 (5)	77 (10)	90 (8)	
First visit	no	1424 (44)	761 (24)	1012 (32)	chi^2^ = 54.19 (<0.001)
	yes	39 (20)	71 (37)	84 (43)	
Attendance length; years (M; SD)	-	7.28 (4.76)	6.59 (4.44)	6.95 (4.33)	5.71 (<0.001) H > M; H > L; M = L *
Attendance last year (M; SD)	-	1.93 (0.67)	1.68 (0.82)	1.58 (0.80)	74.36 (<0.001) H > M; H > L; M > L *
Waiting time from the registration (M; SD)	-	1.93 (0.99)	2.41 (1.33)	1.99 (0.96)	58.08 (<0.001) H < M; H = L; M > L *
Difficulty in registration (M; SD)	-	2.30 (0.83)	2.43 (0.95)	2.72 (0.78)	76.99 (<0.001) H < M; H < L; M < L *
Length of a visit; minutes (M; SD)	-	21.10 (7.63)	20.16 (6.83)	20.00 (7.51)	7.94 (<0.001) H > M; H > L; M = L *
SVS global (M; SD)	-	6.18 (0.79)	5.70 (0.73)	4.72 (0.85)	1051.40 (<0.001) H > M; H > L; M > L *

SRH—self-rated health; HIA—health impact on activities of daily living; SVS—satisfaction with a visit. * Between-group comparisons with T2 Tamhane test.

**Table 3:** Differences between health proactivity profiles according to pre-visit expectations and visit experiences.

	H-pro	M-pro	L-pro	F	Post Hoc Test *
**Pre-Visit Expectations**					
Disease Explanation	6.58 (0.82)	6.40 (0.74)	6.47 (0.81)	14.7 (<0.001)	H > M; H > L; M = L
Treatment Explanation	6.52 (0.81)	6.23 (0.83)	6.40 (0.74)	34.38 (<0.001)	H > M; H > L; M < L
Emotional Support	6.39 (0.97)	5.91 (1.15)	6.34 (0.90)	64.43 (<0.001)	H > M; H = L; M < L
Health Promotion	6.30 (1.02)	5.50 (1.09)	6.11 (1.01)	39.34 (<0.001)	H > M; H > L; M < L
Quality of Life	5.62 (1.74)	5.06 (1.79)	5.30 (1.78)	28.15 (<0.001)	H > M; H > L; M < L
Rapport	6.58 (0.61)	6.23 (0.77)	6.32 (0.73)	78.27 (<0.001)	H > M; H > L; M < L
Communication Skills	6.42 (0.63)	5.94 (0.71)	6.11 (0.79)	137.84 (<0.001)	H > M; H > L; M < L
**Post-Visit Experiences**					
Disease Explanation	6.14 (0.93)	5.68 (0.81)	4.51 (0.96)	1018.87 (<0.001)	H > M; H > L; M = L
Treatment Explanation	5.97 (1.08)	5.60 (0.88)	4.43 (1.1)	708.36 (<0.001)	H > M; H > L; M > L
Emotional Support	5.49 (1.04)	5.54 (0.90)	4.13 (1.02)	692.88 (<0.001)	H = M; H > L; M > L
Health Promotion	5.42 (1.06)	5.43 (1.03)	4.23 (1.05)	482.44 (<0.001)	H = M; H > L; M > L
Quality of Life	4.63 (1.63)	4.84 (1.54)	2.84 (1.38)	550.87 (<0.001)	H < M; H > L; M > L
Rapport	5.88 (0.81)	5.74 (0.82)	4.92 (1.25)	325.65 (<0.001)	H > M; H > L; M > L
Communication Skills	6.03 (0.76)	5.45 (0.71)	4.43 (0.82)	1352.98 (<0.001)	H > M; H > L; M > L
**Post-Pre: Discrepancy between Expectations and Experiences**					
Disease Explanation	−0.44 (0.85)	−0.72 (1.01)	−1.95 (1.15)	772.18 (<0.001)	H > M; H > L; M > L
Treatment Explanation	−0.54 (0.97)	−0.62 (1.09)	−1.96 (1.23)	596.70 (<0.001)	H = M; H > L; M > L
Emotional Support	−0.89 (1.29)	−0.37 (1.42)	−2.20 (1.30)	511.24 (<0.001)	H < M; H > L; M > L
Health Promotion	−0.87 (1.23)	−0.47 (1.35)	−1.88 (1.22)	338.33 (<0.001)	H < M; H > L; M > L
Quality of Life	−0.99 (1.76)	−0.22 (2.09)	−2.46 (1.98)	349.42 (<0.001)	H < M; H > L; M > L
Rapport	−0.69 (0.95)	−0.49 (1.01)	−1.40 (1.21)	212.22 (<0.001)	H < M; H > L; M > L
Communication Skills	−0.39 (.68)	−0.48 (0.84)	−1.69 (0.94)	891.86 (<0.001)	H > M; H > L; M > L

* Between-group comparisons with T2 Tamhane test.

## Data Availability

Data supporting reported results can be obtained from the authors on request.

## Supplementary Materials

The following supporting information can be downloaded at: https://www.mdpi.com/article/10.3390/ijerph19042487/s1, Questionnaires used in the study (Patient booklet).
