# Supplementary material for: Profiles of Health-Related Patient Activation and Their Determinants: The Results of a Cluster Analysis of Older Adults—Conclusions for Patient Counselling
Source: Int J Environ Res Public Health. 2022 Feb 21;19(4):2487. doi: 10.3390/ijerph19042487 (PMC8875668; doi:10.3390/ijerph19042487)
Supplement: Supplementary file 1 [file ijerph-19-02487-s001.zip › ijerph-1557613-supplementary.pdf]

The Patient’s Needs Scale – Pre visit (PNSPre)

Below, you will find a list of statements referring to various elements of a visit at a doctor’s office. Under each statement there is a 7-point response scale. **Please mark with an X the number that describes best, how important to you is a particular element of the visit**. Number 1 indicates that it is completely irrelevant for you, and number 7 – that it is very relevant. We ask for honest answers for all questions.

During this visit it’s important to me, that the doctor …

1. … finds the cause of my symptoms.

1☐ ------- 2☐ ------- 3☐ ------- 4☐ ------- 5☐ ------- 6☐ ------- 7☐

Completely irrelevant *Very relevant*

2. … tells me about the probable course of my treatment.

1☐ ------- 2☐ ------- 3☐ ------- 4☐ ------- 5☐ ------- 6☐ ------- 7☐

Completely irrelevant *Very relevant*

3. … explains possible consequences of illness.

1☐ ------- 2☐ ------- 3☐ ------- 4☐ ------- 5☐ ------- 6☐ ------- 7☐

Completely irrelevant *Very relevant*

During this visit it’s important to me, that the doctor …

1. … presents the results of my tests.

1☐ ------- 2☐ ------- 3☐ ------- 4☐ ------- 5☐ ------- 6☐ ------- 7☐

Completely irrelevant *Very relevant*

2. … gives me some advice about medications I’m taking.

1☐ ------- 2☐ ------- 3☐ ------- 4☐ ------- 5☐ ------- 6☐ ------- 7☐

Completely irrelevant *Very relevant*

3. … presents recommendations for the treatment.

1☐ ------- 2☐ ------- 3☐ ------- 4☐ ------- 5☐ ------- 6☐ ------- 7☐

Completely irrelevant *Very relevant*

During this visit it’s important to me, that the doctor …

1. … talks with me about how I feel and how I cope.

1☐ ------- 2☐ ------- 3☐ ------- 4☐ ------- 5☐ ------- 6☐ ------- 7☐

Completely irrelevant *Very relevant*

2. … gives me reassurance.

1☐ ------- 2☐ ------- 3☐ ------- 4☐ ------- 5☐ ------- 6☐ ------- 7☐

Completely irrelevant *Very relevant*

3. … shows me his care.

1☐ ------- 2☐ ------- 3☐ ------- 4☐ ------- 5☐ ------- 6☐ ------- 7☐

Completely irrelevant *Very relevant*

COPY

© Zespół PRACTA, WUM/NTNU

During this visit it's important to me, that the doctor …

1. … talks with me about what harms my health.

1☐ ------- 2☐ ------- 3☐ ------- 4☐ ------- 5☐ ------- 6☐ ------- 7☐

Completely irrelevant *Very relevant*

2. … suggests what I can do to improve my functioning in everyday life.

1☐ ------- 2☐ ------- 3☐ ------- 4☐ ------- 5☐ ------- 6☐ ------- 7☐

Completely irrelevant *Very relevant*

3. … encourages me to make health-promoting changes (i.e. proper diet, physical activity).

1☐ ------- 2☐ ------- 3☐ ------- 4☐ ------- 5☐ ------- 6☐ ------- 7☐

Completely irrelevant *Very relevant*

During this visit it's important to me, that the doctor …

4. … suggests how to maintain social relationships.

1☐ ------- 2☐ ------- 3☐ ------- 4☐ ------- 5☐ ------- 6☐ ------- 7☐

Completely irrelevant *Very relevant*

5. … talks with me about how to spend time actively.

1☐ ------- 2☐ ------- 3☐ ------- 4☐ ------- 5☐ ------- 6☐ ------- 7☐

Completely irrelevant *Very relevant*

6. … suggests ways of maintaining life satisfaction.

1☐ ------- 2☐ ------- 3☐ ------- 4☐ ------- 5☐ ------- 6☐ ------- 7☐

Completely irrelevant *Very relevant*

During this visit it's important to me, that the doctor …

1. … is benevolent towards me.

1☐ ------- 2☐ ------- 3☐ ------- 4☐ ------- 5☐ ------- 6☐ ------- 7☐

Completely irrelevant *Very relevant*

2. … treats me seriously.

1☐ ------- 2☐ ------- 3☐ ------- 4☐ ------- 5☐ ------- 6☐ ------- 7☐

Completely irrelevant *Very relevant*

3. … shows me respect.

1☐ ------- 2☐ ------- 3☐ ------- 4☐ ------- 5☐ ------- 6☐ ------- 7☐

Completely irrelevant *Very relevant*

COPY

Communication Scale – Pre visit (CSPre)

Below you'll find a list of various behaviors of a doctor during a visit. Like previously, **we ask to mark with an X the square with a number that is the closest to your opinion how relevant for you are particular actions/behaviors of a doctor during the visit.**

It's important for me that the doctor …	*Completely irrelevant* … *Very relevant*
1. … greets me in a kind manner.	1☐ ---- 2☐ ---- 3☐ ---- 4☐ ---- 5☐ ---- 6☐ ---- 7☐
2. … discusses with me the reason of a visit.	1☐ ---- 2☐ ---- 3☐ ---- 4☐ ---- 5☐ ---- 6☐ ---- 7☐
3. … listens to me carefully.	1☐ ---- 2☐ ---- 3☐ ---- 4☐ ---- 5☐ ---- 6☐ ---- 7☐
4. … shows understanding for my problems.	1☐ ---- 2☐ ---- 3☐ ---- 4☐ ---- 5☐ ---- 6☐ ---- 7☐
5. … makes sure he understood me correctly.	1☐ ---- 2☐ ---- 3☐ ---- 4☐ ---- 5☐ ---- 6☐ ---- 7☐
6. … encourages me to ask questions.	1☐ ---- 2☐ ---- 3☐ ---- 4☐ ---- 5☐ ---- 6☐ ---- 7☐
7. … answers all my questions.	1☐ ---- 2☐ ---- 3☐ ---- 4☐ ---- 5☐ ---- 6☐ ---- 7☐
8. … makes sure if I understood him correctly.	1☐ ---- 2☐ ---- 3☐ ---- 4☐ ---- 5☐ ---- 6☐ ---- 7☐
9. … uses language I can understand.	1☐ ---- 2☐ ---- 3☐ ---- 4☐ ---- 5☐ ---- 6☐ ---- 7☐
10. … summarizes topics we've discussed.	1☐ ---- 2☐ ---- 3☐ ---- 4☐ ---- 5☐ ---- 6☐ ---- 7☐

It's important for me that the doctor …	*Completely irrelevant* … *Very relevant*
11. … informs me about the examination.	1☐ ---- 2☐ ---- 3☐ ---- 4☐ ---- 5☐ ---- 6☐ ---- 7☐
12. … cares about my comfort during the examination (i.e. measuring blood pressure).	1☐ ---- 2☐ ---- 3☐ ---- 4☐ ---- 5☐ ---- 6☐ ---- 7☐
13 … provides as much time as I need for each part of the visit(the interview, preparation for the examination, thinking, etc.).	1☐ ---- 2☐ ---- 3☐ ---- 4☐ ---- 5☐ ---- 6☐ ---- 7☐

	It's important for me that the doctor …	*Completely irrelevant* — *Very relevant*
14	… explains treatment options available in my situation	1☐ ---- 2☐ ---- 3☐ ---- 4☐ ---- 5☐ ---- 6☐ ---- 7☐
15.	… explains why I should comply to the presented recommendations.	1☐ ---- 2☐ ---- 3☐ ---- 4☐ ---- 5☐ ---- 6☐ ---- 7☐
16.	… makes sure I'll be able to comply to the recommendations.	1☐ ---- 2☐ ---- 3☐ ---- 4☐ ---- 5☐ ---- 6☐ ---- 7☐
17.	… writes down main recommendations for me (medications, dosage, etc).	1☐ ---- 2☐ ---- 3☐ ---- 4☐ ---- 5☐ ---- 6☐ ---- 7☐
18.	… discusses the plan of further treatment.	1☐ ---- 2☐ ---- 3☐ ---- 4☐ ---- 5☐ ---- 6☐ ---- 7☐
19.	… summarizes briefly the entire visit.	1☐ ---- 2☐ ---- 3☐ ---- 4☐ ---- 5☐ ---- 6☐ ---- 7☐

	It's important for me that the doctor …	*Completely irrelevant* — *Very relevant*
1.	… encourages me to participate in making decisions.	1☐ ---- 2☐ ---- 3☐ ---- 4☐ ---- 5☐ ---- 6☐ ---- 7☐
2.	… gives me opportunity to express my opinion.	1☐ ---- 2☐ ---- 3☐ ---- 4☐ ---- 5☐ ---- 6☐ ---- 7☐
3.	… takes my opinion into account in making decisions.	1☐ ---- 2☐ ---- 3☐ ---- 4☐ ---- 5☐ ---- 6☐ ---- 7☐
4.	… creates the atmosphere that allows to discuss intimate issues freely.	1☐ ---- 2☐ ---- 3☐ ---- 4☐ ---- 5☐ ---- 6☐ ---- 7☐
5.	… notices my feelings and accepts them.	1☐ ---- 2☐ ---- 3☐ ---- 4☐ ---- 5☐ ---- 6☐ ---- 7☐
6.	… takes care of a good atmosphere during the entire visit.	1☐ ---- 2☐ ---- 3☐ ---- 4☐ ---- 5☐ ---- 6☐ ---- 7☐
7.	… wins my trust.	1☐ ---- 2☐ ---- 3☐ ---- 4☐ ---- 5☐ ---- 6☐ ---- 7☐
		Completely irrelevant — *Very relevant*

© Zespół PRACTA, WUM/NTNU

General information

1. Age: ______________

2. Gender: F ☐ M ☐

3. Marital status:

☐ single
☐ partnership/marriage
☐ separated/divorced
☐ widowed

4. Education:

☐ primary
☐ voccational
☐ secondary (without matura exam)
☐ secondary (with matura exam)
☐ higher

5. Place of residence:

☐ rural area
☐ small town (up to 20 thousands. citizens)
☐ medium town (21-100 thousands. citizens)
☐ large town (101-500 thousands citizens)
☐ Warsaw
☐ other city (above 500 thousands citizens)

6. Who do you live with? [*one or more „X”*]

☐ alone
☐ with a spouse/partner
☐ with children
☐ with grandchildren
☐ with other members of my family
☐ with other persons, not members of the family
☐ in the old people's home

7. What is your professional status? [*one or more „X”*]

☐ professionally active – working full time
☐ professionally active – part time
☐ retired
☐ annuitant
☐ unemployed

8. What is your financial situation?

☐ poor ☐ rather poor ☐ average ☐ rather good ☐ good

COPY

Health status

1. How do you evaluate your health? (*in comparison with people of similar age*)

☐ very good ☐ good ☐ average ☐ poor ☐ very poor

2. Have you attended within past 6 months: [*one or more „X"*]:

<u>In the hospital</u>

☐ Emergency room, without need to stay in the hospital

☐ hospital for a surgery or other procedure

☐ hospital for treatment or observation (other than a surgery)

<u>On an outpatient bases</u>

☐ other General Practitioner

☐ a specialist

☐ general medical tests (blood count, ECG, etc.)

☐ sanatorium

☐ none of the above

3. How many diseases have you had/have you been currently treated for?

☐none ☐1 disease ☐2-3 diseases ☐ 4-5 diseases ☐6 or more disesases

4. Please specify to what extent your health limits your ability to maitain the following: [*one„X" for each statement]*:

	Doesn't limit at all	*Limits a little*	*Limits moderately*	*Limits very much*
Full body hygene	1☐ ----------	2☐ ----------	3☐ ----------	4☐
Dressing	1☐ ----------	2☐ ----------	3☐ ----------	4☐
Preparing and eating meals	1☐ ----------	2☐ ----------	3☐ ----------	4☐
Medication taking	1☐ ----------	2☐ ----------	3☐ ----------	4☐
Moving around the house	1☐ ----------	2☐ ----------	3☐ ----------	4☐
Reading and watching TV	1☐ ----------	2☐ ----------	3☐ ----------	4☐
Shopping	1☐ ----------	2☐ ----------	3☐ ----------	4☐
Commuting	1☐ ----------	2☐ ----------	3☐ ----------	4☐
Driving	1☐ ----------	2☐ ----------	3☐ ----------	4☐
Dealing with official matters	1☐ ----------	2☐ ----------	3☐ ----------	4☐

COPY

© Zespół PRACTA, WUM/NTNU

Today’s visit to the doctor

When answering questions below, please refer to the today’s visit to the doctor.

1. How long have you been attending this doctor?
   ☐ it was my first visit
   ☐ I attend longer, please specify number of years ________

2. How often have you attended this doctor within last year?
   ☐ not at all ☐ 1 – 2 times ☐ 3 – 7 times ☐ 8 or more times

3. How long have your been waiting for this visit since you’ve made an appointment?
   ☐ visit on the same day
   ☐ up to 3 days
   ☐ 4 – 7 days
   ☐ 1 to 2 weeks
   ☐ more than 2 weeks
   ☐ I don’t remember

4. How easy was it to schedule a visit to this doctor?
   ☐ very easy ☐ easy ☐ moderately easy ☐ difficult ☐ very difficult

5. What is the aim of your today’s visit? [*one or more „X”*]
   ☐ asking for advice
   ☐ treatment (i.e. prescribing medicines, referring to tests, etc.)
   ☐ referral to a specialist
   ☐ referral to medical tests
   ☐ check-up
   ☐ signing documents
   ☐ other

COPY

TIME FOR A VISIT TO THE DOCTOR

The Patient's Satisfaction with a Visit Scale- Post visit (SVSPost)

1. Would you recommend this doctor to your family/friends?

1☐ ------- 2☐ ------- 3☐ ------- 4☐ ------- 5☐ ------- 6☐ ------- 7☐

definitely no ***definitely yes***

2. Would you like to come to this doctor again?

1☐ ------- 2☐ ------- 3☐ ------- 4☐ ------- 5☐ ------- 6☐ ------- 7☐

definitely no ***definitely yes***

3. If it was easy to change a clinic – would you still come to this one?

1☐ ------- 2☐ ------- 3☐ ------- 4☐ ------- 5☐ ------- 6☐ ------- 7☐

definitely no ***definitely yes***

1. How satisfied are you wiht this visit at the doctor?

1☐ ------- 2☐ ------- 3☐ ------- 4☐ ------- 5☐ ------- 6☐ ------- 7☐

definitely dissatisfied ***definitely satisfied***

2. Have your hopes for this visit been fulfilled?

1☐ ------- 2☐ ------- 3☐ ------- 4☐ ------- 5☐ ------- 6☐ ------- 7☐

definitely no ***definitely yes***

3. How satisfied are you with the time the doctor has spent on the consultation?

1☐ ------- 2☐ ------- 3☐ ------- 4☐ ------- 5☐ ------- 6☐ ------- 7☐

definitely dissatisfied ***definitely satisfied***

4. Considering registration, travel, waiting time, help you received, etc., was the visit worth coming for?

1☐ ------- 2☐ ------- 3☐ ------- 4☐ ------- 5☐ ------- 6☐ ------- 7☐

definitely no ***definitely yes***

How long have you been waiting in the waiting room for this visit?

☐less than 15 minutes ☐ approximately half and hour ☐1 hour or longer ☐don't remember

COPY

The Patient's Needs Scale – Post visit (PNSPost)

During this visit the doctor …

1. … found the cause of my symptoms.

   1☐ ------- 2☐ ------- 3☐ ------- 4☐ ------- 5☐ ------- 6☐ ------- 7☐
   Completely irrelevant *Very relevant*

2. … presented me the probable course of my treatment.

   1☐ ------- 2☐ ------- 3☐ ------- 4☐ ------- 5☐ ------- 6☐ ------- 7☐
   Completely irrelevant *Very relevant*

3. … explained possible consequences of illness.

   1☐ ------- 2☐ ------- 3☐ ------- 4☐ ------- 5☐ ------- 6☐ ------- 7☐
   Completely irrelevant *Very relevant*

During this visit the doctor …

1. … presented the results of my tests.

   1☐ ------- 2☐ ------- 3☐ ------- 4☐ ------- 5☐ ------- 6☐ ------- 7☐
   Completely irrelevant *Very relevant*

2. … gave me some advice about medications I take.

   1☐ ------- 2☐ ------- 3☐ ------- 4☐ ------- 5☐ ------- 6☐ ------- 7☐
   Completely irrelevant *Very relevant*

3. … presented recommendations for my treatment.

   1☐ ------- 2☐ ------- 3☐ ------- 4☐ ------- 5☐ ------- 6☐ ------- 7☐
   Completely irrelevant *Very relevant*

During this visit the doctor …

1. … talked to me about how I felt and how I coped.

   1☐ ------- 2☐ ------- 3☐ ------- 4☐ ------- 5☐ ------- 6☐ ------- 7☐
   Completely irrelevant *Very relevant*

2. … gave me reassurance.

   1☐ ------- 2☐ ------- 3☐ ------- 4☐ ------- 5☐ ------- 6☐ ------- 7☐
   Completely irrelevant *Very relevant*

3. … showed me his care.

   1☐ ------- 2☐ ------- 3☐ ------- 4☐ ------- 5☐ ------- 6☐ ------- 7☐
   Completely irrelevant *Very relevant*

COPY

During this visit the doctor …

1. … talked with me about what harme my health.

1☐ ------- 2☐ ------- 3☐ ------- 4☐ ------- 5☐ ------- 6☐ ------- 7☐

Completely irrelevant — *Very relevant*

2. … suggested what I can do to improve my functioning in everyday life.

1☐ ------- 2☐ ------- 3☐ ------- 4☐ ------- 5☐ ------- 6☐ ------- 7☐

Completely irrelevant — *Very relevant*

3. … encouraged me to make health-promoting changes (i.e. proper diet, physical activity).

1☐ ------- 2☐ ------- 3☐ ------- 4☐ ------- 5☐ ------- 6☐ ------- 7☐

Completely irrelevant — *Very relevant*

During this visit the doctor …

1. … suggested how to maintain social relationships.

1☐ ------- 2☐ ------- 3☐ ------- 4☐ ------- 5☐ ------- 6☐ ------- 7☐

Completely irrelevant — *Very relevant*

2. … talked with me about how to spend time actively.

1☐ ------- 2☐ ------- 3☐ ------- 4☐ ------- 5☐ ------- 6☐ ------- 7☐

Completely irrelevant — *Very relevant*

3. … suggested ways of maintaining life satisfaction.

1☐ ------- 2☐ ------- 3☐ ------- 4☐ ------- 5☐ ------- 6☐ ------- 7☐

Completely irrelevant — *Very relevant*

During this visit the doctor …

1. … was benevolent towards me.

1☐ ------- 2☐ ------- 3☐ ------- 4☐ ------- 5☐ ------- 6☐ ------- 7☐

Completely irrelevant — *Very relevant*

2. … treated me seriously.

1☐ ------- 2☐ ------- 3☐ ------- 4☐ ------- 5☐ ------- 6☐ ------- 7☐

Completely irrelevant — *Very relevant*

3. … showed me respect.

1☐ ------- 2☐ ------- 3☐ ------- 4☐ ------- 5☐ ------- 6☐ ------- 7☐

Completely irrelevant — *Very relevant*

COPY

Communication Scale – Post Visit (CSPost)

During this visit the doctor …	*Definitely yes*						*Definitely no*
1. … greeted me in a kind manner.	1☐ ----	2☐ ----	3☐ ----	4☐ ----	5☐ ----	6☐ ----	7☐
2. … discussed with me the reason of a visit.	1☐ ----	2☐ ----	3☐ ----	4☐ ----	5☐ ----	6☐ ----	7☐
3. … listened to me carefully.	1☐ ----	2☐ ----	3☐ ----	4☐ ----	5☐ ----	6☐ ----	7☐
4. … showed understanding for my problems.	1☐ ----	2☐ ----	3☐ ----	4☐ ----	5☐ ----	6☐ ----	7☐
5. … made sure he understood me correctly.	1☐ ----	2☐ ----	3☐ ----	4☐ ----	5☐ ----	6☐ ----	7☐
6. … encouraged me to ask questions.	1☐ ----	2☐ ----	3☐ ----	4☐ ----	5☐ ----	6☐ ----	7☐
7. … answered all my questions.	1☐ ----	2☐ ----	3☐ ----	4☐ ----	5☐ ----	6☐ ----	7☐
8. … made sure if I understood him correctly.	1☐ ----	2☐ ----	3☐ ----	4☐ ----	5☐ ----	6☐ ----	7☐
9. … used language I could understand.	1☐ ----	2☐ ----	3☐ ----	4☐ ----	5☐ ----	6☐ ----	7☐
10. … summarized topics we had discussed.	1☐ ----	2☐ ----	3☐ ----	4☐ ----	5☐ ----	6☐ ----	7☐

During this visit the doctor …	*Definitely yes*						*Definitely no*
11. … informed me about the examination.	1☐ ----	2☐ ----	3☐ ----	4☐ ----	5☐ ----	6☐ ----	7☐
12. … cared about my comfort during the examination (i.e. measuring blood pressure).	1☐ ----	2☐ ----	3☐ ----	4☐ ----	5☐ ----	6☐ ----	7☐
13 … provided as much time as I needed for each part of the visit(the interview, preparation for the examination, thinking, etc.).	1☐ ----	2☐ ----	3☐ ----	4☐ ----	5☐ ----	6☐ ----	7☐

© Zespół PRACTA, WUM/NTNU

During this visit the doctor …		Definitely yes						Definitely no
14	… explained treatment options available in my situation	1☐	2☐	3☐	4☐	5☐	6☐	7☐
15.	… explained why I should comply to the presented recommendations.	1☐	2☐	3☐	4☐	5☐	6☐	7☐
16.	… made sure I would be able to comply to the recommendations.	1☐	2☐	3☐	4☐	5☐	6☐	7☐
17.	… wrote down main recommendations for me (medications, dosage, etc).	1☐	2☐	3☐	4☐	5☐	6☐	7☐
18.	… discussed the plan of further treatment.	1☐	2☐	3☐	4☐	5☐	6☐	7☐
19.	… summarized briefly the entire visit.	1☐	2☐	3☐	4☐	5☐	6☐	7☐

During this visit the doctor …		Definitely yes						Definitely no
1.	… encouraged me to participate in making decisions.	1☐	2☐	3☐	4☐	5☐	6☐	7☐
2.	… gave me opportunity to express my opinion.	1☐	2☐	3☐	4☐	5☐	6☐	7☐
3.	… took my opinion into account in making decisions.	1☐	2☐	3☐	4☐	5☐	6☐	7☐
4.	… created the atmosphere that allowed to discuss intimate issues freely.	1☐	2☐	3☐	4☐	5☐	6☐	7☐
5.	… noticed my feelings and accepted them.	1☐	2☐	3☐	4☐	5☐	6☐	7☐
6.	… took care of a good atmosphere during the entire visit.	1☐	2☐	3☐	4☐	5☐	6☐	7☐
7.	… won my trust.	1☐	2☐	3☐	4☐	5☐	6☐	7☐
		Definitely yes						Definitely no

© Zespół PRACTA, WUM/NTNU

The Attitude Toward Illness and Treatment Scale – Post (ATITSPost)

Statements below refer to your today's visit to the doctor. Please, mark with an X the number on the scale from 1 to 7, which describes your response best.

Due to this visit at the doctor …

5. … I understand the nature and causes of my ailments.

1☐ ------- 2☐ ------- 3☐ ------- 4☐ ------- 5☐ ------- 6☐ ------- 7☐

definitely no ***definitely yes***

6. … I know available treatment options for my ailments.

1☐ ------- 2☐ ------- 3☐ ------- 4☐ ------- 5☐ ------- 6☐ ------- 7☐

definitely no ***definitely yes***

7. … I understand the results of all my medical tests.

1☐ ------- 2☐ ------- 3☐ ------- 4☐ ------- 5☐ ------- 6☐ ------- 7☐

definitely no ***definitely yes***

8. … I know how each of my medications works.

1☐ ------- 2☐ ------- 3☐ ------- 4☐ ------- 5☐ ------- 6☐ ------- 7☐

definitely no ***definitely yes***

9. … I know how to prevent further problems with my health.

1☐ ------- 2☐ ------- 3☐ ------- 4☐ ------- 5☐ ------- 6☐ ------- 7☐

definitely no ***definitely yes***

10. … I know how to sustain or improve health according to my age.

1☐ ------- 2☐ ------- 3☐ ------- 4☐ ------- 5☐ ------- 6☐ ------- 7☐

definitely no ***definitely yes***

Due to this visit at the doctor …

11. … I'll be able to cope with the treatment and compliance.

1☐ ------- 2☐ ------- 3☐ ------- 4☐ ------- 5☐ ------- 6☐ ------- 7☐

definitely no ***definitely yes***

12. … I think I can influence how I'll feel in the future.

1☐ ------- 2☐ ------- 3☐ ------- 4☐ ------- 5☐ ------- 6☐ ------- 7☐

definitely no ***definitely yes***

13. … I understand that my active participation in treatment and disease prevention is important for my functioning and performance.

1☐ ------- 2☐ ------- 3☐ ------- 4☐ ------- 5☐ ------- 6☐ ------- 7☐

definitely no ***definitely yes***

COPY

Due to this visit at the doctor …

1. … I feel calmer.

1☐ ------- 2☐ ------- 3☐ ------- 4☐ ------- 5☐ ------- 6☐ ------- 7☐

definitely no *definitely yes*

2. … I believe the treatment will be effective.

1☐ ------- 2☐ ------- 3☐ ------- 4☐ ------- 5☐ ------- 6☐ ------- 7☐

definitely no *definitely yes*

3. … I hope my health will improve.

1☐ ------- 2☐ ------- 3☐ ------- 4☐ ------- 5☐ ------- 6☐ ------- 7☐

definitely no *definitely yes*

Due to this visit at the doctor …

1. … I feel depressed.

1☐ ------- 2☐ ------- 3☐ ------- 4☐ ------- 5☐ ------- 6☐ ------- 7☐

definitely no *definitely yes*

2. … I have fears about my symptoms.

1☐ ------- 2☐ ------- 3☐ ------- 4☐ ------- 5☐ ------- 6☐ ------- 7☐

definitely no *definitely yes*

3. … I doubt that improvement in my functioning is possible.

1☐ ------- 2☐ ------- 3☐ ------- 4☐ ------- 5☐ ------- 6☐ ------- 7☐

definitely no *definitely yes*

Due to this visit at the doctor …

1. … I'm going to comply to the recommendations conscientiously.

1☐ ------- 2☐ ------- 3☐ ------- 4☐ ------- 5☐ ------- 6☐ ------- 7☐

definitely no *definitely yes*

2. … I'm going to find out more about my health state.

1☐ ------- 2☐ ------- 3☐ ------- 4☐ ------- 5☐ ------- 6☐ ------- 7☐

definitely no *definitely yes*

3. … I'm making a plan how to comply to the recommendations.

1☐ ------- 2☐ ------- 3☐ ------- 4☐ ------- 5☐ ------- 6☐ ------- 7☐

definitely no *definitely yes*

4. … I'm going to participate in the treatment actively.

1☐ ------- 2☐ ------- 3☐ ------- 4☐ ------- 5☐ ------- 6☐ ------- 7☐

definitely no *definitely yes*

COPY

COPY
